# A real-world comparison study of the efficacy of dual-target first-line rescue treatment of human epidermal growth factor receptor 2 positive advanced breast cancer: trastuzumab combined with pertuzumab versus trastuzumab combined with pyrotinib

**DOI:** 10.3389/fonc.2026.1720851

**Published:** 2026-03-04

**Authors:** Liang Zhang, Chao Li, Shubin Song, Fukai Wang, Tingting Ding, Zhengrui Liu, Yuqin Jin, Zhiyong Yu

**Affiliations:** 1 Department of Breast Surgery, Shandong Cancer Hospital and Institute Shandong First Medical University and Shandong Academy of Medical Sciences, Jinan, China; 2 Department of Imaging, Shandong Cancer Hospital and Institute Shandong First Medical University and Shandong Academy of Medical Sciences, Jinan, China

**Keywords:** advanced breast cancer, first-line therapy, human epidermal growth factor receptor 2, monoclonal antibody, tyrosine kinase inhibitor

## Abstract

**Introduction:**

Trastuzumab combined with pertuzumab (HP) is a first-line therapy for advanced breast cancer (ABC). However, trastuzumab combined with pyrotinib (HPyr) could also exert complementary and synergistic effects. Currently, clinical trials directly comparing the effectiveness of the above two treatment approaches are lacking. Herein, a registered single-center, retrospective study (NCT04609540) was caried out.

**Methods:**

In the present study, patients diagnosed with human epidermal growth factor receptor 2 (HER2)-positive ABC and treated with dual-target first-line rescue treatment at the Shandong Cancer Hospital between January 2018 and February 2023 were included. Patients were assigned to the HP or HPyr treatment groups by the physician-in-charge. The clinical, pathological and prognostic data of all patients were collected and recorded.

**Results:**

Among the 89 patients included, 47 received HP, while the remaining 42 HPyr. The therapeutic effect of each treatment approach was determined via evaluating progression-free survival (PFS). The results showed that patients who were treated with HPyr displayed a higher progression rate (71.4% vs. 63.8%) compared with those treated with HP. However, statistical significance was not reached (mean PFS, 21.0±1.9 vs. 24.1±2.8 months; P=0.653). In addition, patients of >60 years old, who received HPyr and younger patients (≤40 years old) who received HP had longer PFS (22.8±4.9 vs. 16.8±4.2 months; P=0.332; and 27.4±5.5 vs. 15.8±5.6 months; P=0.098, respectively). PFS without significant differences was also obtained in the other subgroups. Furthermore, HP showed better clinical efficacy in young patients compared with older ones, while HPyr benefitted older patients. In the other subgroups, the two dual-target regimens also displayed curative effects, without significant differences.

**Discussion:**

Overall, the results of the current study suggested that HPyr could be equivalently used as HP, as a first-line treatment strategy for patients with HER2-positive ABC. Currently, more prospective large-sample studies are needed to further validate our conclusions.

## Introduction

Breast cancer is the most common type of cancer and the most serious death threat for female cancer patients (1). The human epidermal growth factor receptor 2 (HER2) gene was first discovered in 1987 (2). It has been reported that HER2 upregulation accounts for approximately 15-20% of all breast cancer cases, while it is used to predict early recurrence, metastasis, and poor prognosis (3). Currently, targeted treatment strategies for HER2-positive breast cancer include specific monoclonal antibodies, such as trastuzumab and pertuzumab, and tyrosine kinase inhibitors (TKIs), including lapatinib, lenvatinib and pyrotinib.

Trastuzumab is the first monoclonal antibody which specifically binds to the extracellular segment of HER2. It inhibits tumor growth via inhibiting the dimerization of HER2 and other HERs, and is therefore applied to improve the survival of patients for more than 10 years (4). However, due to the development of drug resistance, HER2-positive metastatic breast cancer still threatens the lives of patients due to the development of drug resistance. In recent years, several drug resistance mechanisms, such as impaired drug binding to HER2, constitutive activation of signaling pathways parallel to or downstream of HER2, metabolic reprogramming, and decreased activation of the immune system, have been identified (5).

Pertuzumab, a HER2-directed humanized monoclonal antibody, exerts different binding sites from those of trastuzumab. Pertuzumab inhibits the two main signaling pathways of ligand initiated intracellular signaling, namely mitogen activated protein kinase and phosphoinositol 3-kinase signaling, thus leading to cell growth arrest and apoptosis. Pyrotinib is a pan-HER TKI that targets HER1, HER2, and HER4. It can inhibit tyrosine kinase phosphorylation via irreversibly binding to the ATP binding site on the intracellular tyrosine kinase region, thus blocking subsequent signal transduction (6, 7). Compared with monoclonal antibodies, TKIs have the advantages of multiple targets, low cardiotoxicity, and convenient oral administration (8).

In theory, dual target therapy of trastuzumab combined with pyrotinib (HPyr) is a promising combination for HER2 inhibition, since the underlying mechanisms are different than those previously known. It has been also reported that this regimen is characterized by the lack of adverse reactions. The macromolecule binds to the extracellular HER2 domain, while micromolecular TKIs penetrate the cell membrane to target the HER family’s membrane domain. They complement each other internally and externally.

Currently, trastuzumab combined with pertuzumab (HP) is considered as a first-line rescue treatment strategy for advanced breast cancer (ABC) (9) However, HPyr could achieve complementary and synergistic effects. Nowadays, comparative clinical trial evidence on the effectiveness of HPyr compared with HP is still missing. Therefore, we retrospectively analyzed the efficacy of HP compared with that of HPyr as first-line rescue treatment of patients with HER2-positive ABC patients.

## Materials and methods

### Inclusion and exclusion criteria

The current single-center, retrospective study included patients, who were diagnosed with HER2-positive ABC and received first-line rescue treatment at the Shandong Cancer Hospital between January 2018 and February 2023. The study was conducted according to the Chinese laws and regulatory requirements. The inclusion criteria were as follows: i. Patients with pathological confirmation of breast cancer, clinically diagnosed as ABC, and without rescue treatment; ii. immunohistochemistry results showing HER2 3+ or HER2 2+ with a positive fluorescence *in situ* hybridization test; and iii. patients who received HP or HPyr targeted therapy. The exclusion criteria were the following: i. Patients who received targeted therapy other than trastuzumab, pertuzumab or pyrotinib; ii. those who were treated with single targeted therapy; and iii. those who did not receive targeted therapy; or received rescue therapy in the past.

### HER2 assessment

HER2 testing was performed using the methodology outlined in the American Society of Clinical Oncology (ASCO)/CAP HER2 testing guidelines. The therapeutic effect of each treatment approach was evaluated using the Response Evaluation Criteria in Solid Tumors (10). Tumor progression was defined as the appearance of new lesions or an increase in tumor diameter (or the sum of all lesion diameters) by more than 20% in imaging compared with the previous examination.

### Grouping

Patients were assigned to the HP or HPyr by the supervising physician. In addition, chemotherapy and endocrine treatment strategies were also developed by the supervising physician. The initial dose of trastuzumab was 8mg/kg, followed by 6mg/kg of each 21-day cycle. Patients who were treated with HPyr were given continuous oral pyrotinib at an initial dose of 400 mg once daily. Based on patient tolerance, the dose could be reduced to 320 and 240 mg per day. In the HP group, the initial dose of pertuzumab was 840mg, followed by 420 mg of each 21-day cycle until disease progression. The clinical, pathological, and prognostic data of all patients were collected and recorded in this study. For premenopausal patients, endocrine therapy involves aromatase inhibitors (AIs, including Exemestane, Letrozole, and Anastrozole) in combination with ovarian function suppression (OFS), while for postmenopausal patients, endocrine therapy consists of AIs alone.

### Statistical analysis

The SPSS software version 22 (SPSS, Chicago, IL, USA) was used for all statistical analyses. A P value of < 0.05 for a two-sided test was considered statistically significant. Continuous data are expressed as medians and intervals, while categorical ones are expressed as counts and percentages. The therapeutic effect was determined via evaluating progression-free survival (PFS). Survival analysis was calculated by Kaplan-Meier analysis.

## Results

### Patient characteristics

A total of 89 eligible patients were enrolled, including 47 and 42 patients in the HP and HPyr groups, respectively. All patients were female, with an average onset age of 50.4 years. Except for the family history of cancer and brain metastases, the baseline characteristics were generally well balanced between the two groups (**Table 1**). Two brain metastases in HPyr group were active, and the other 8 brain metastases in both groups were stable. One patient had bilateral breast cancer. The average and median follow-up time for patients in the HP group was 29.1 ± 11.2 months and 34 months, while that for patients in the HPyr group was 28.6 ± 8.9 months and 35 months.

**Table 1:** Baseline characteristics of patients.

Variable	HP (n=47)	HPyr (n=42)	P value
Family history of cancer	3	6	0.011
Onset age	50.6 (26–75)	50.2 (31–71)	0.068
<40	10	5	
41-50	13	15	
51-60	15	18	
>60	9	4	
Primary tumor side			0.319
Left	24	20	
Right	22	22	
Initial diagnosis status			0.406
Advanced stage	29	18	
Early stage	18	24	
Stage I	0	4	
Stage II a	6	3	
Stage II B	3	4	
Stage III A	4	6	
Stage III B	–	–	
Stage III C	5	7	
Menstrual status			0.955
Postmenopausal	27	18	
Premenopausal	20	24	
Hormone receptor expression			0.482
Positive	22	18	
Negative	25	24	
Viscera metastases	33	33	0.074
Brain metastases	1	9	<0.001
Liver metastases	15	18	0.054
Lung metastases	24	20	0.816
Bone metastases	21	17	0.440
Palliative surgery			0.486
Yes	9	7	
No	38	35	
Combined therapy			
Initial chemotherapy	47	42	0.069
T	22	25	
X	5	7	
N	3	2	
AC-T	5	4	
TCb	12	4	
Maintenance therapy	43	32	0.264
X	19	15	
N	3	2	
AI ± OFS	21	15	
Response Evaluation			0.133
Progressive Disease	30 (63.8%)	30 (71.4%)	
Stable Disease	4	3	
Partial Response	10	7	
Complete Response	3	2	
Survival			0.295
Yes	43	37	
No	4	5	

HPyr, trastuzumab combined with pyrotinib; HP, trastuzumab combined with pertuzumab; T, Docetaxel or Nab-paclitaxel; X, Capecitabine; N, Vinorelbine; Cb, Carboplatin; A, anthracyclines; C, Cyclophosphamide; AI, Aromatase inhibitor; OFS, Ovarian Function Suppression.

All patients continued to receive dual-targeted therapy until disease progression. The most common and severe adverse reaction was myelosuppression, which was resolved with supportive care. Although some patients required chemotherapy dose reductions, no treatment was discontinued due to myelosuppression. Other manageable adverse reactions included joint stiffness, limb numbness, nausea, vomiting, and alopecia (**Table 2**).

**Table 2:** Median cycles of treatment and adverse events.

Variable	HP (n=47)	HPyr (n=42)
Cycles of treatment	Median cycles (range)	Median cycles (range)
Docetaxel	6 (3 – 6)	6(2 - 6)
Nab-paclitaxel (weekly)	18(6 - 18)	18(12 - 18)
H	17(2 - 52)	20.5(3 - 48)
P	17(2 - 52)	
Pyr		20.5(3 - 48)
Adverse Events		
TEAE	44	41
Grade ≥r TEAE	3	14
Drug dose reduction	2	6

H, trastuzumab; P, pertuzumab; Pyr, pyrotinib; TEAE, treatment-emergent adverse events;.

### HP and HPyr therapy, and PFS

**Figure 1:**
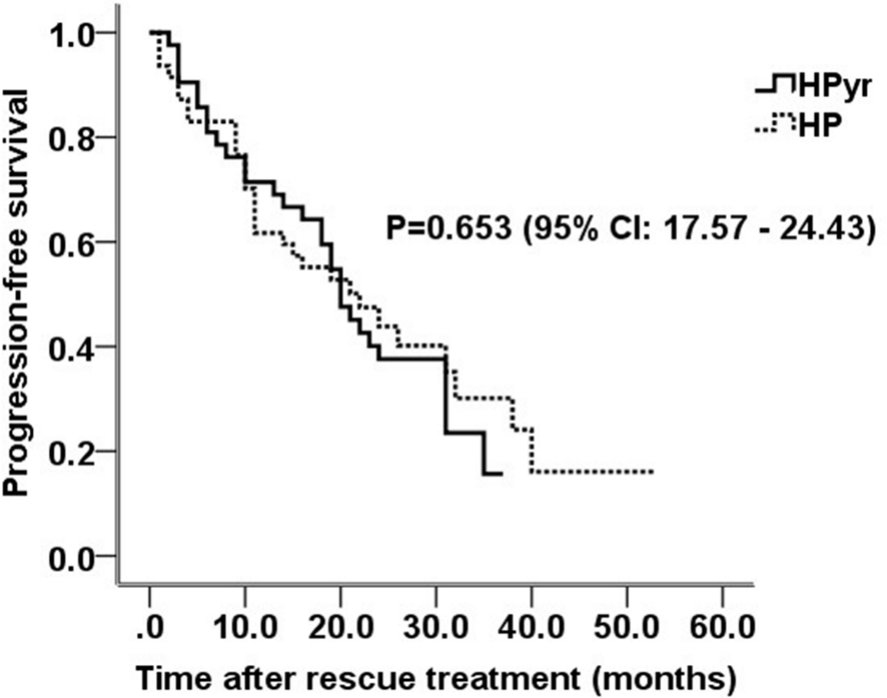
Survival curves for all patients in HP and HPyr groups. HPyr, trastuzumab combined with pyrotinib; HP, trastuzumab combined with pertuzumab; CI, confidence interval.

**Figure 2:**
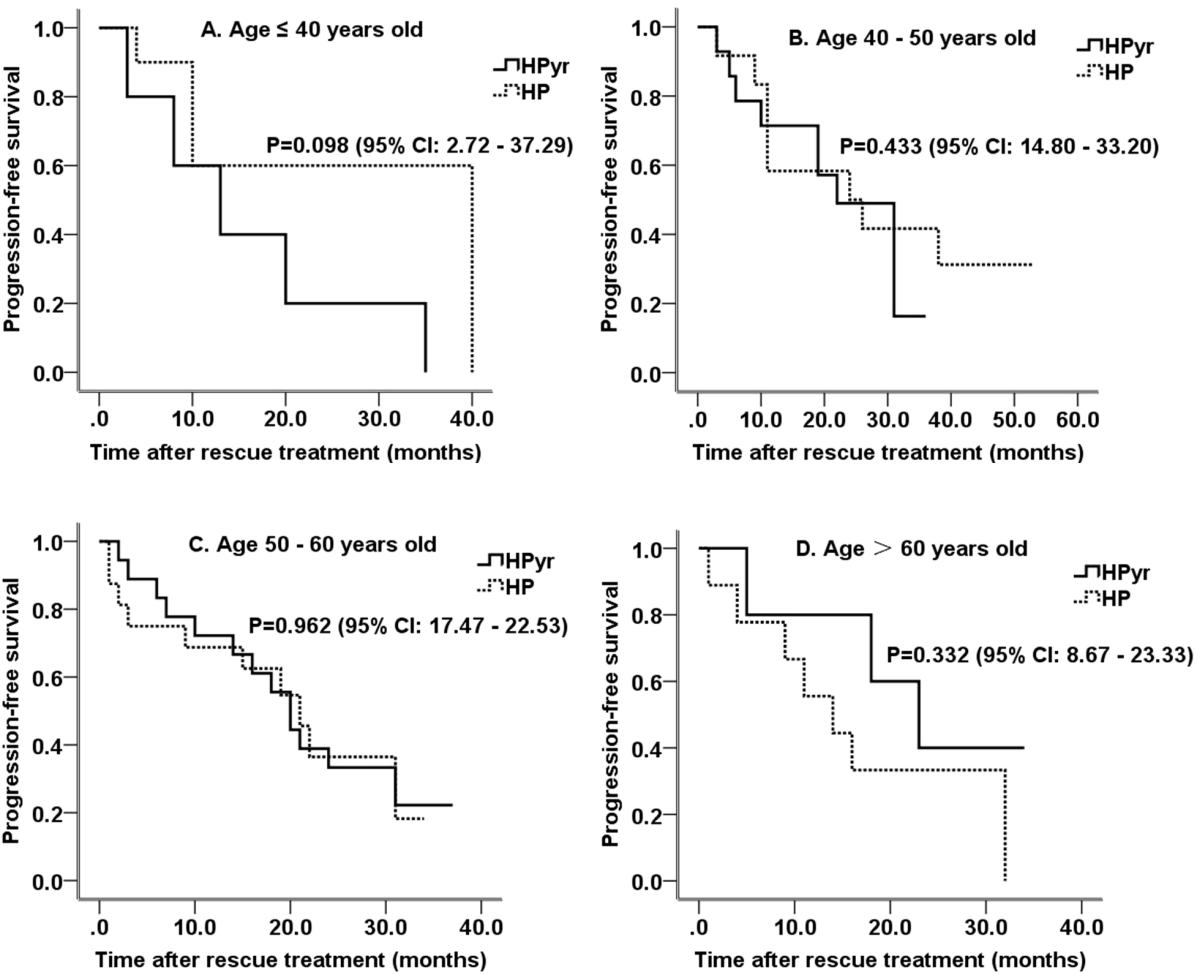
Survival curves of patients in different age brackets. **(A)** Survival curves for patients ≤40 years old; **(B)** Survival curves for patients over 40 years old but ≤50 years old; **(C)** Survival curves for patients over 50 years old but ≤60 years old; **(D)** Survival curves for patients over 60 years old. HPyr, trastuzumab combined with pyrotinib; HP, trastuzumab combined with pertuzumab; CI, confidence interval.

The average PFS time was 23.3 ± 2.0 months, with a median PFS of 21.0 ± 1.8 months. Overall, patients who received HPyr dual-target therapy had a larger progression rate (71.4% vs. 63.8%). However, there was no significant difference in PFS between the HPyr and HP groups (mean PFS, 21.0 ± 1.9 vs. 24.1 ± 2.8 months; P = 0.653; 95% confidence interval (CI), 17.57-24.43; **Figure 1**). Older patients (>60 years old) who received HPyr therapy showed a longer PFS (22.8 ± 4.9 vs. 16.8 ± 4.2 months; P = 0.332; 95% CI, 8.67-23.33; **Figure 2D**). Additionally, younger patients (≤ 40 years old) who were treated with HP also displayed a longer PFS (27.4 ± 5.5 vs. 15.8 ± 5.6 months; P = 0.098; 95% CI, 2.72-37.29; **Figure 2A**). However, statistical significance was not reached. For patients aged 40–50 and 50–60 years, there was no significant difference in PFS between the two groups (P = 0.433; 95% CI, 14.80-33.20 and P = 0.962; 95% CI, 17.47-22.53, respectively; **Figure 2B, C**). Consistently, PFS efficacy was also achieved in various different subgroups based on the primary tumor side, initial diagnosis status, menstrual status, hormone receptor expression, visceral metastasis site, and whether palliative surgery was performed (**Table 3**). There was only one patient with brain metastasis in the HP group, and therefore an efficient analysis could not be performed.

**Table 3:** Progression-free survival of patients.

Variable	HP (month)	HPyr (month)	P value
Primary tumor side			
Left	27.1 ± 4.4	19.8 ± 2.6	0.461
Right	22.1 ± 3.1	22.0 ± 2.6	0.834
Initial diagnosis status			
Advanced stage	23.8 ± 3.5	19.2 ± 2.3	0.392
Early stage	22.3 ± 3.9	22.0 ± 2.7	0.942
Menstrual status			
Postmenopausal	19.8 ± 2.6	21.6 ± 2.3	0.838
Premenopausal	28.7 ± 4.7	20.4 ± 2.7	0.339
Hormone receptor expression			
Positive	25.3 ± 3.1	19.3 ± 2.7	0.158
Negative	22.2 ± 4.0	22.2 ± 2.6	0.543
Viscera metastases	22.0 ± 3.5	19.8 ± 2.0	0.883
Liver metastases	21.4 ± 5.0	19.4 ± 2.6	0.959
Lung metastases	19.0 ± 3.3	17.8 ± 2.4	0.919
Bone metastases	24.7 ± 4.4	21.0 ± 2.7	0.643
Palliative surgery			
Yes	33.1 ± 6.7	27.4 ± 2.1	0.365
No	20.8 ± 2.5	19.1 ± 2.0	0.538

HPyr, trastuzumab combined with pyrotinib; HP, trastuzumab combined with pertuzumab.

### Palliative surgery and PFS

**Figure 3:**
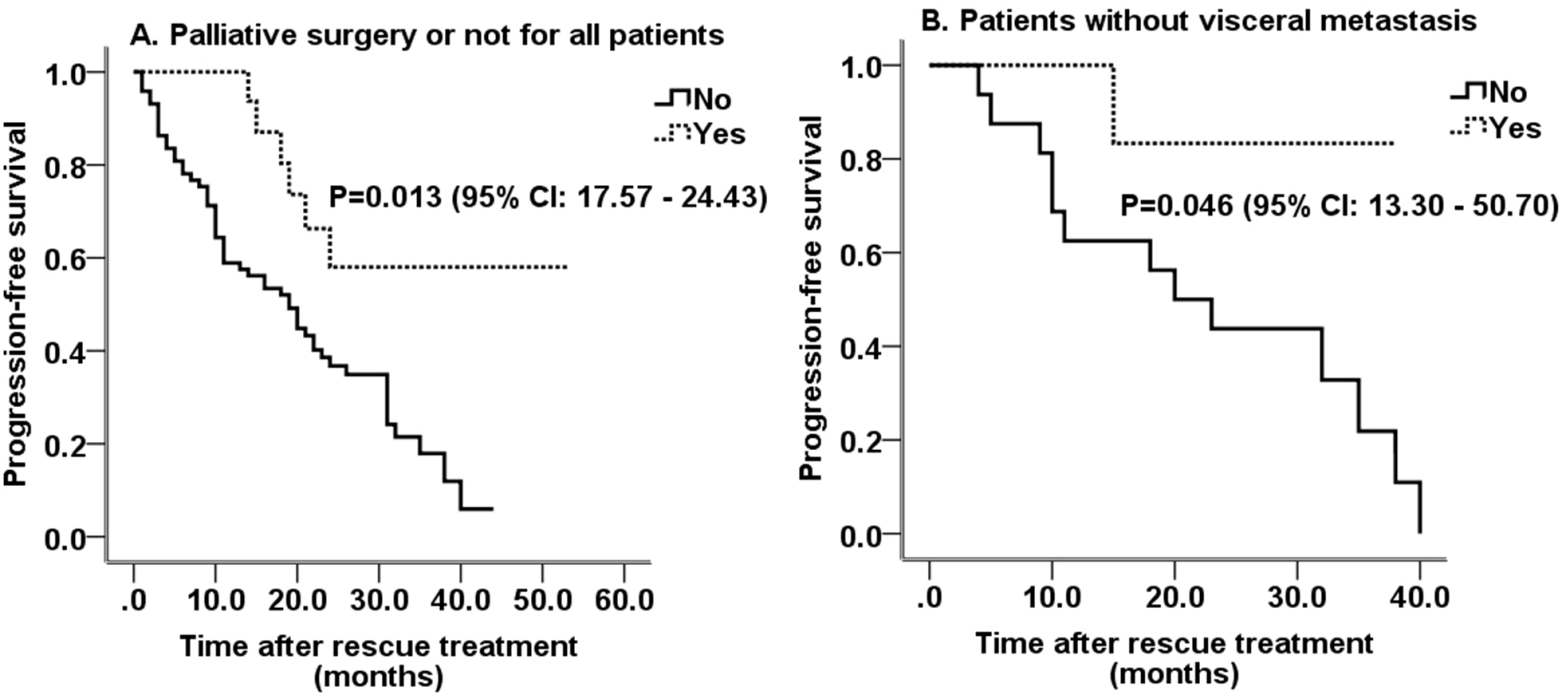
Survival curves of patients underwent palliative surgery or not. **(A)** Survival curves for all patients underwent palliative surgery or not; **(B)** Survival curves for patients without visceral metastasis underwent palliative surgery or not. HPyr, trastuzumab combined with pyrotinib; HP, trastuzumab combined with pertuzumab; CI, confidence interval.

A total of 16 patients underwent palliative surgery, including nine with visceral metastasis, six with liver metastasis, four with lung metastasis, and five with bone metastasis. No brain metastasis was recorded prior surgery. In addition, patients who underwent palliative surgery showed a significantly longer PFS compared with those who did not (38.6 ± 4.5 vs. 20.1 ± 1.7 months; P = 0.013; 95% CI, 17.57-24.43; **Figure 3A**). Palliative surgery still improved PFS in patients without visceral metastasis (34.2 ± 3.5 vs. 22.7 ± 3.4; P = 0.046 (34.2 ± 3.5 vs. 22.7 ± 3.4 months; P = 0.046; 95% CI, 13.30-50.70; **Figure 3B**).

## Discussion

Compared with single-target therapy, dual-target therapy combined with chemotherapy (with or without endocrine therapy for hormone receptor-positive patients) has been proven to improve the survival of patients in the first-line rescue treatment of HER2-positive ABC and is therefore considered as the standard treatment approach for ABC (11–14). The CLEOPATRA trial (13) showed that docetaxel combined with HP significantly improved overall survival by 15.7 months (56.5 vs. 40.8 months; P<0.001) compared to docetaxel combined with trastuzumab. Additionally, this therapeutic strategy notably extended PFS by 6 months, thus establishing the first-line standard treatment status of the HP regimen.

During the development of HER2-positive ABC, brain metastasis occurs in ~30-50% of patients and is the main cause of treatment failure, thus ultimately leading to shortened survival (15). Compared with macromolecular monoclonal antibodies, micromolecular TKIs, such as pyrotinib, are more likely to penetrate the blood-brain barrier, effectively control brain metastasis, prolong patient survival (8) and improve patient sensitivity to cranial radiation therapy (16). The PERMEATE trial (15) showed that for patients with HER2-positive ABC with brain metastasis, who did not undergo local radiotherapy, pyrotinib combined with capecitabine, yielded a better intracranial objective response rate (74.6%) and median PFS (11.3 months) compared with other agents. The PHENIX (17), PHOEBE (18), and PHILA (19) trials showed that pyrotinib was associated with a good response rate for extracranial lesions and was of practical clinical significance in reducing the risk of brain metastasis and delaying occurrence.

The first-line rescue efficacy of HP compared with that of HPyr has not been previously validated. However, the comparisons of the data between different studies could provide novel insights into the efficacy of these treatment approaches. The PERTAIN trial (20) indicated that the PFS benefit of HP and aromatase inhibitors (AIs) was superior to that of trastuzumab and AIs, particularly for patients not receiving chemotherapy, for whom the median PFS was 22.6 months. However, dual-target therapy did not improve PFS (16.9 vs. 16.9 months) in patients who received induction chemotherapy (21), thus suggesting that HP could only benefit some high-risk patients.

The PHILA trial (19) showed that the median PFS in patients treated with docetaxel combined with HPyr group was 24.3 months, which was significantly better than that in the docetaxel combined with trastuzumab group(PFS, 10.4 months). This was the longest survival without progression in first-line treated HER2-positive ABC patients reported thus far, with an absolute difference of 14 months. This difference was greater compared with the difference of 6 months reported in the CLEOPATRA trial. However, different studies cannot be directly compared. This is also evident from the data on risk ratios. Therefore, the risk ratio in the HPyr combined with docetaxel group was 0.41, while that in the HP combined with docetaxel group was 0.62. The former exerted a numerical advantage. On the other hand, the data from different clinical studies also corroborated each other. The PHILA trial was not the only superior validation study. Moreover, pyrotinib displayed a stable therapeutic effect on HER2-positive breast cancer both as a second-line and late-line treatment (PHOEBE and PHENIX trials), and as a new adjuvant treatment (PHEDRA, NeoATP, and PANPHILA trials).

Currently there is no study to prospectively compare HP vs. HPyr, but the current retrospective study showed that HP was superior for patients <40 years of age, while HPyr was more effective for patients >60 years of age. However, the difference was not significant. In addition, there was no significant difference in the efficacy of the HP and HPyr dual targets for first-line rescue of ABC in the other subgroups. Based on the current medical insurance policy for the treatment of ABC in China, pertuzumab cannot be reimbursed, while pyrotinib is included in the medical insurance reimbursement list. As a domestically produced drug in China that can be reimbursed, pyrotinib is cheaper than pertuzumab and has better treatment accessibility. Furthermore, pertuzumab is administrated through intravenous infusion, while pyrotinib is administered orally. Therefore, the dual-target HPyr is more easily accepted by patients in terms of economy, accessibility and convenience.

Herein, whether palliative surgery was necessary for patients receiving first-line rescue treatment was also analyzed. Therefore, palliative surgery could improve PFS in all patients, including those without visceral metastasis. On the one hand, this difference may be related to the reduction in tumor burden, and on the other hand, the metastasis burden was more limited in patients who underwent surgery. Therefore, for patients with extensive metastasis, the therapeutic value and surgical approach selection of palliative surgery deserve further discussion.

The present study has certain limitations. Most of the comparative results in this study did not show statistical differences, since the number of patients was relatively small. Therefore, the research conclusions require a larger sample size or additional multivariate analysis to be further confirmed and clarified. Herein, a comparison based on brain metastasis could not be performed, since there was only one patient with brain metastasis in the HP group. Rescue chemotherapy and endocrine treatment plans are not unique. This can cause certain interference in the evaluation of therapeutic effects. Due to the short follow-up time, effective overall survival statistical analysis could not be conducted, which was a significant limitation for evaluating the treatment effectiveness in advanced cancer patients. PFS data can provide evidence of “disease control ability” but cannot answer the core question of “whether it prolongs survival.” In addition, as a retrospective study, this study only received feedback from a small number of patients with severe toxic side effects in this regard, and effective statistical analysis could not be performed. Therefore, these findings need to be further clarified in prospective clinical trials. However, there is still a lack of information on whether such clinical trials have been established.

## Conclusion

From the retrospective non-randomized small study, HP showed better clinical efficacy as a first-line rescue treatment for ABC in younger patients, while the efficacy of HPyr was numerically better in older patients. Currently, more prospective large-sample studies are needed to further validate our conclusions.

## Data Availability

The original contributions presented in the study are included in the article/supplementary material. Further inquiries can be directed to the corresponding author.
